# Radiographic Outcome of Surgical Treatment of Scheuermann’s Kyphosis: a Comparative Study Between Old and New Spinal Instruments

**DOI:** 10.5812/ircmj.2621

**Published:** 2013-04-05

**Authors:** Farzad Omidi-Kashani, Mohamed H Ebrahimzadeh, Ali Birjandinejad

**Affiliations:** 1 Orthopaedic Research Center, Emam Reza Hospital, Mashhad University of Medical Sciences, Mashhad, IR Iran

**Keywords:** Scheuermann Disease, Surgical Procedures, Operative, Therapeutics

**Dear Editor,**

Scheuermann’s kyphosis (SK) is the most common cause of structural kyphosis in adolescents (1). Surgery appears to be the only way to significantly improve the kyphotic deformity associated with SK. The need for the anterior procedure can be questioned when adequate correction and fusion can be achieved by a posterior procedure alone using pedicular screws with or without Smith-Petersen osteotomies (2, 3).

The aim of this retrospective study was to evaluate and compare the radiographic outcomes of surgery in patients with Scheuermann’s kyphosis operated by old (Harrington Instrumentation; HI) and new segmental spinal implants (Diapason instrumentation; Stryker; DI). Our inclusion criteria were unsightly appearance, painful or progressive curves refractory to brace treatment, or curve magnitude greater than 65°. The patients with other causes of kyphosis or follow-up period less than 2 years were excluded. From October 1971 to November 1998, 37 cases with HI (group A) and from then to August 2008 33 cases with DI fulfilled these criteria.

We routinely performed two stage surgeries except in the cases that kyphosis decreased to 40° in supine lateral hyperextension view. Fusion levels are determined from the standing anteroposterior and lateral radiographs (3). The operative technique was according to the standard surgical manner (4). The Cobb method was used to measure the curve magnitude (5). Data were analyzed using SPSS software (version 18, Chicago, IL, USA). The paired t test was used to compare pre- and postoperative kyphosis while independent t test was used to compare mean loss of correction. P value equal to or below 0.05 was considered statistically significant. Our patients’ characteristics are depicted in Table 1. All the patients except one (in Group A) treated with combined anterior and posterior approaches.

**Table 1.** Two Groups Treated With Harrington and Diapason Instrumentation

Implant	HI^a^	DI^b^
**Cases**	37	33
**Age, year ± SD**	20.2 ± 3.7	20.5 ± 4.3
**Sex No. (SD)** **^c^**		
Male	24 (1.85)	14 (0.74)
Female	13 (1.85)	19 (0.74)
**Mean preoperative kyphosis, Mean ± SD**		
Standing	90.7 ± 8.8	88 ± 9.2
Stress	64 ± 9.7	60.9 ± 10.9
**Mean postoperative kyphosis, Mean ± SD**		
Immediate	41.7 ± 8.5	45.2 ± 6.3
Final	47.6 ± 9.6	48.5 ± 6.4
**Mean immediate correction, Mean ± SD**	49 ± 7.6	42.8 ± 8.4
**Mean Loss of Correction, Mean ± SD**	6 ± 3.3	3.2 ± 1.4
**Mean Follow up, year ± SD**	8.2 ± 3.7	4.5 ± 1.1

^a^Harrington Instrumentation

^b^Diapason Instrumentation

^c^Standard Deviation

Recently, most studies concerning the surgical treatment of SK showed better results, especially in adults, when a combined anterior and posterior approach was used (3, 6-8). The modern instrumentation systems provide a more rigid fixation, and postoperative bracing is no longer necessary (9, 10).

This study shows that although immediate kyphosis correction was higher in Harrington group (P = 0.0019), throughout the time loss of correction was also higher in this group (P < 0.0001). A lesser amount of correction that was obtained in Group B could be due to the fact that with increasing concept of the normal spinal alignment, more vigorous correction was not necessary because this could be potentially harmful. Therefore less correction should not be judged as incapability. Furthermore with modern spinal instrumentation, the amount of correction is much more durable and associated with less radiologic complications.

## References

[1] Lowe TG, Line BG (2007). Evidence based medicine: analysis of Scheuermann kyphosis.. Spine (Phila Pa 1976)..

[2] Papagelopoulos PJ, Klassen RA, Peterson HA, Dekutoski MB (2001). Surgical treatment of Scheuermann's disease with segmental compression instrumentation.. Clin Orthop Relat Res..

[3] Lim M, Green DW, Billinghurst JE, Huang RC, Rawlins BA, Widmann RF (2004). Scheuermann kyphosis: safe and effective surgical treatment using multisegmental instrumentation.. Spine (Phila Pa 1976)..

[4] Arlet V, Schlenzka D (2005). Scheuermann's kyphosis: surgical management.. Eur Spine J..

[5] Cobb JR (1948). Outline for the study of scoliosis.. Instr Course Lect..

[6] Jansen RC, van Rhijn LW, van Ooij A (2006). Predictable correction of the unfused lumbar lordosis after thoracic correction and fusion in Scheuermann kyphosis.. Spine (Phila Pa 1976)..

[7] Lowe TG, Kasten MD (1994). An analysis of sagittal curves and balance after Cotrel-Dubousset instrumentation for kyphosis secondary to Scheuermann's disease. A review of 32 patients.. Spine (Phila Pa 1976)..

[8] Speck GR, Chopin DC (1986). The surgical treatment of Scheuermann's kyphosis.. J Bone Joint Surg Br..

[9] Damborg F, Engell V, Andersen M, Kyvik KO, Thomsen K (2006). Prevalence, concordance, and heritability of Scheuermann kyphosis based on a study of twins.. J Bone Joint Surg Am..

[10] Papagelopoulos PJ, Mavrogenis AF, Savvidou OD, Mitsiokapa EA, Themistocleous GS, Soucacos PN (2008). Current concepts in Scheuermann's kyphosis.. Orthopedics..

